# Osteoid osteoma of the scapula mimicking a pathology of the shoulder

**DOI:** 10.11604/pamj.2017.26.94.11692

**Published:** 2017-02-24

**Authors:** Zeineb Alaya, Walid Osman

**Affiliations:** 1 Department of Rheumatology, Farhat Hached Hospital, Faculty of Medicine of Sousse, Sousse, Tunisia; 2 Department of Orthopaedics, Sahloul Hospital, Faculty of Medicine of Sousse, Sousse, Tunisia

**Keywords:** Osteoid osteoma, CT scan, bone scintigraphy, MRI, bone tumor

## Image in medicine

A 14-year-old patient presented with chronic shoulder pain evolving since three years. The pain persisted despite the various medicated and physical treatments followed. The clinical examination noted a normal shoulder examination with pain facing the right scapula without local inflammatory signs. There were no abnormalities in the biological examinations, in particular absence of biological inflammatory syndrome, rheumatoid factor negative and normal calcemia. Radiographs of the shoulder and scapula were normal. Bone scintigraphy showed hyperfixation of the radiotracer regarding the right scapular spine evoking an osteoid osteoma (A). CT scan of the right scapula showed a very limited osteolytic lesion without osteocondensation edges with a non-enhanced hypodense matrix after injection of contrast agent (B). MRI showed lesion of the right scapular spine in hypersignal T1 and T2 without cortical rupture or periosteal reaction suggestive of a benign lesion such as osteoid osteoma (C, D). The patient had a biopsy with surgical resection of the lesion. The diagnosis of osteoid osteoma was confirmed histologically. The peculiarity of this observation lies in the rarity of this localization and the deceptive clinical picture suggesting a shoulder pathology in first intention. In cases of diagnostic doubt and in the presence of normal radiographs, the most specific examination is thin-section CT scans, the sensitivity of which can be improved by association with an MRI.

**Figure 1:**
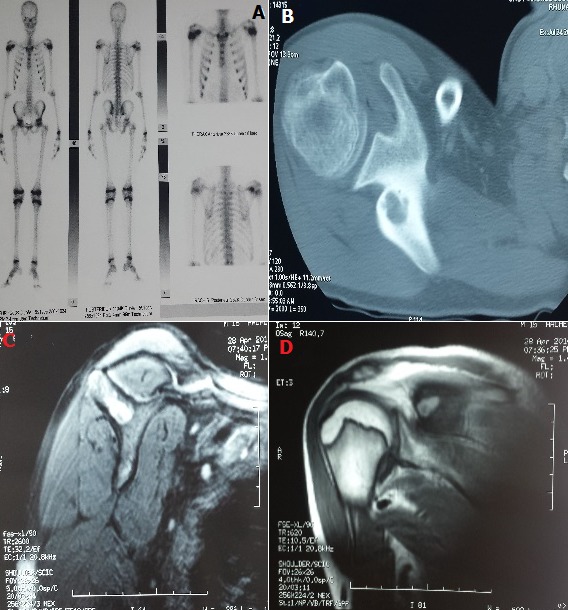
A) bone scintigraphy: hyperfixation of the radiotracer regarding the right scapular spine evoking an osteoid osteoma; B) CT scan of the right scapula: a very limited osteolytic lesion without osteocondensation edges with a non-enhanced hypodense matrix after injection of contrast agent; (C,D) MRI of the scapula: lesion of the right scapular spine in hypersignal T1 and T2 without cortical rupture or periosteal reaction suggestive of a benign lesion such as osteoid osteoma

